# Effect of Combined Physical and Cognitive Interventions on Executive Functions in OLDER Adults: A Meta-Analysis of Outcomes

**DOI:** 10.3390/ijerph17176166

**Published:** 2020-08-25

**Authors:** Wei Guo, Ming Zang, Sebastian Klich, Adam Kawczyński, Małgorzata Smoter, Biye Wang

**Affiliations:** 1 College of Physical Education, Yangzhou University, Yangzhou 225009, China; guowei@yzu.edu.cn (W.G.); zangming@yzu.edu.cn (M.Z.); 2 Institute of Sports, Exercise and Brain, Yangzhou University, Yangzhou 225009, China; 3 Department of Paralympic Sport, University School of Physical Education in Wrocław, 51-617 Wrocław, Poland; sebastian.klich@awf.wroc.pl (S.K.); adam.kawczynski@awf.wroc.pl (A.K.); 4 Diagnostic and Rehabilitation Center ‘Promyk Słońca’, 50-088 Wrocław, Poland; m.h.smoter@gmail.com

**Keywords:** combined physical and cognition interventions, executive functions, meta-analysis, older adults

## Abstract

*Background:* Both physical exercise and cognitive training can effectively improve executive functions in older adults. However, whether physical activity combined with cognitive training is more effective than a single intervention remains controversial. The aim of this study was to perform a meta-analysis to evaluate the effect of combined physical and cognitive interventions on executive functions in older adults aged 65–80 years old. *Methods:* Randomized controlled trials of combined physical and cognitive interventions on executive functions in older adults were searched using the Web of Science, Elsevier Science, PubMed, EBSCO, Springer-Link, and NATURE databases. Data extraction and quality evaluation were done by Comprehensive Meta-Analysis, V3. *Results:* A total of 21 studies were included. The results showed that the combined physical and cognitive interventions produced significantly larger gains in executive functions, compared to the control group (standardized mean difference (SMD) = 0.26, 95% confidence interval (CI) [0.14, 0.39], *p* < 0.01). Furthermore, the effects of the combined physical and cognitive interventions were moderated by the study quality, intervention length, and intervention frequency. No significant differences were found between the combined interventions and the physical intervention alone (SMD = 0.13, 95% CI [−0.07, 0.33], *p* > 0.05) or the cognitive intervention alone (SMD = 0.13, 95% CI [−0.05, 0.30], *p* > 0.05). *Conclusions:* The combined physical and cognitive interventions effectively delayed the decrease of executive functions in older adults and this effect was influenced by the length and frequency of the intervention as well as the research quality. However, the effect of the combined physical and cognitive interventions was not significantly better than that of each intervention alone.

## 1. Introduction

Executive functions (EFs), also known as executive control or cognitive control, refers to a set of top-down mental processes [1]. The framework of EFs, which is divided into response inhibition, complex EFs, set-shifting, and updating, has been widely accepted [2,3,4,5]. It has been proven that the decline of EFs in older adults can seriously affect their daily lives and is the main cause of cognitive aging [6,7]. Therefore, it is important to counteract the decline of EFs in older adults. Fortunately, some methods have been found to delay this process. Physical activities are recommended as an effective nonpharmacological approach to improve EFs in healthy older adults [8,9,10,11]. In addition, a recent meta-analysis has reported that physical activities can effectively improve the EFs of older adults with mild cognitive impairment [12]. Meanwhile, cognitive training is another type of behavioral intervention that has been demonstrated to effectively delay cognitive aging. Examples of cognitive training include playing video games [13,14], television-based cognitive training [15], computerized cognitive training [16,17], playing board games [18,19], and mathematical training [20]. In terms of the efficacy of cognitive training in older adults with mild cognitive impairment, studies published over the past decade have revealed that cognitive training can effectively enhance specific cognitive functions, such as attention, orientation, verbal fluency, visual memory, etc. [21,22,23].

Based on the effect and mechanism revealed by previous studies, it is assumed that the combination of cognitive training and physical exercise may bring greater cognitive benefits than physical exercise or cognitive training alone [24,25,26,27]. Physical exercise can enhance cell proliferation and synaptic plasticity and cognitive training can guide and integrate new neurons and synapses into existing neural networks; therefore, the combination of the two interventions may induce superimposed cognitive benefits [28,29]. However, whether such a benefit exists remains controversial in older adults with or without cognitive impairment [26,27,30]. A systematic review has revealed an additional cognitive benefit of the combined interventions in healthy older adults but not in older adults with cognitive impairment [31]. In contrast, recent randomized controlled trials have shown that the combination of physical exercise and cognitive training can also improve cognitive functions in older adults with cognitive impairment [32,33].

Most of the studies mentioned above focused on the beneficial effects of physical exercise and cognitive training on overall cognitive functions, while only a few studies focused on specific cognitive functions, especially EFs. Thus, the main purpose of the present study was to explore whether the combination of cognitive training and physical exercise can effectively delay the decline of EFs in older adults. Furthermore, the current meta-analysis focused on potential moderating variables, such as the mode of combination. Studies have shown that different modes of combination may cause different results. The separation of the combined interventions is a possible cause of the negative result [34]. Meanwhile, simultaneous combined interventions have been demonstrated to induce beneficial effects on cognitive functions in older adults [24,35,36]. The possible reason for this discrepancy is that neural plasticity is influenced by time factors and will return to the baseline level at one hour after exercise. Therefore, the simultaneous combined interventions could obtain more cognitive benefits before returning to baseline [37]. In addition, other crucial variables, including cognitive status, intervention length, intervention frequency, session length, and research quality, were included as moderators.

The present meta-analysis adopted Comprehensive Meta-Analysis software to quantitatively evaluate the effect of the combined interventions on EFs in older adults to compare the effects between the combined intervention group, the physical intervention alone group, the cognitive intervention alone group, and the control group. Furthermore, the moderating effects were explored for all the following variables: intervention plan, research quality, and characteristics of the sample.

## 2. Materials and Methods

### 2.1. Search Strategy

The meta-analysis was conducted in accordance with the Preferred Reporting Items for Systematic Reviews and Meta-Analyses (PRISMA) statement. Systematic computer-based searches of the Web of Science, Elsevier Science, PubMed, and NATURE databases since the start of each database until December 2019 were performed. The following keywords were used: combined intervention terms (“combined” OR “multimodal” OR “dual-task”), AND physical intervention terms (“exercise” OR “physical” OR “training”), AND cognitive intervention terms (“cognitive function” OR “mental training” OR “EFs”), AND aging population terms (“older adult” OR “aged” OR “aging”). The articles in the selected journals were further screened and additional searches were conducted using the same search terms in Google Scholar to identify other potentially relevant articles.

### 2.2. Eligibility Criteria

Studies were considered eligible for this meta-analysis if the following criteria were fulfilled: (1) the study design must be a randomized controlled trial. (2) The sample population must be older adults (healthy or with mild cognitive impairment), but the patients could not have any neurological conditions other than mild cognitive impairment, such as a major depression. The age range had not been pre-determined and eventually resulted from the selected articles. (3) The intervention strategies must include a combination of physical and cognitive training as well as at least one comparison group in the article. (4) The intervention must have been carried out for more than 6 weeks. (5) Enough information must be reported in the article to calculate an effect size for at least one EF outcome measure. (6) The article must be written in English. The following types of studies were excluded: (1) nonintervention studies, (2) unpublished studies, abstracts, or papers, (3) review articles or theoretical articles, (4) those with interventions that were not adequately explained, and (5) the combined intervention group was not compared with any of these following groups: an active/passive control group, a physical intervention alone group, or a cognitive intervention alone group.

### 2.3. Data Extraction and Analysis

Except for the overall EFs, the present meta-analysis also divided EFs into distinguished sub-functions based on the classification used in a recent meta-analysis [38]. The tasks we chose to tap the response inhibition were Flanker task, Stroop test, and letter sets test. All the tasks require participants to suppress an interfering dominant response in order to achieve a specific goal. Set-shifting included trail-making test A and B, switching test, Digit-Symbol Substitution Test, and Dimensional Change Card Sort Test and these tasks require participants to monitor and code the input information related to the current task and replace the old information with the new information. Complex executive function included Self-Ordered Pointing task, Matrix Reasoning Test, Complex Figure Test, Behavioral Assessment of the Dysexecutive Syndrome, Controlled Oral Words Association Test, Groton Maze Learning test, Regensburger Wort Flüssigkeits-Test, and Leistungs-Prüf-System. All of these tasks include a series of components such as planning, reasoning, and problem-solving. The reason for excluding the updating component of EFs was that there were only three studies using updating tasks (N-back task and Subtest Digit Span Backwards test) in the included literature [25,34,39]. The methodology and important trial characteristics, including the study population, intervention type, and comparison of multi-armed intervention groups, were obtained from the articles and listed in a spreadsheet.

For the meta-analysis, EFs outcome data were extracted in the form of means, standard deviations (SDs), and the number of participants of each group at baseline and post-intervention. If the means and SDs were not available, the changes in the mean and SD between, at baseline, and after the intervention were extracted, or the upper and lower limits of the 95% confidence interval (CI) were used to calculate the SDs, and Comprehensive Meta-Analysis software was used for quantitative synthesis. In this study, the combined intervention group was compared with the control group, the cognitive intervention alone group, and the physical intervention alone group, respectively, and the intervention effect was measured by the standardized mean difference (SMD) after comparison. A positive effect size indicates that the combined intervention group has a better intervention effect than the comparison group. After the effect size of each study was obtained, the combined effect size and the 95% CI were calculated to evaluate the effect of the combined interventions on EFs in older adults.

### 2.4. Evaluation of Methodological Quality

The methodological quality of the identified articles was evaluated independently by two reviewers (M.Z. and B.W.). A 13-item checklist modified from the Delphi list was used to assess the methodological quality [40] and any disagreements on ratings were discussed with a third reviewer (W.G.) before a final decision was determined. One point was awarded for each fulfilled criterion on the scale and no points were awarded if the criterion was not met; the quality score for each article ranged from 0 to 13. A total score of 10–13 points was defined as a high methodological quality, a total score of 7–9 points was defined as a medium methodological quality, and a total score of less than 7 points suggested a low methodological quality [31].

## 3. Results

### 3.1. Included Studies

**Figure 1:**
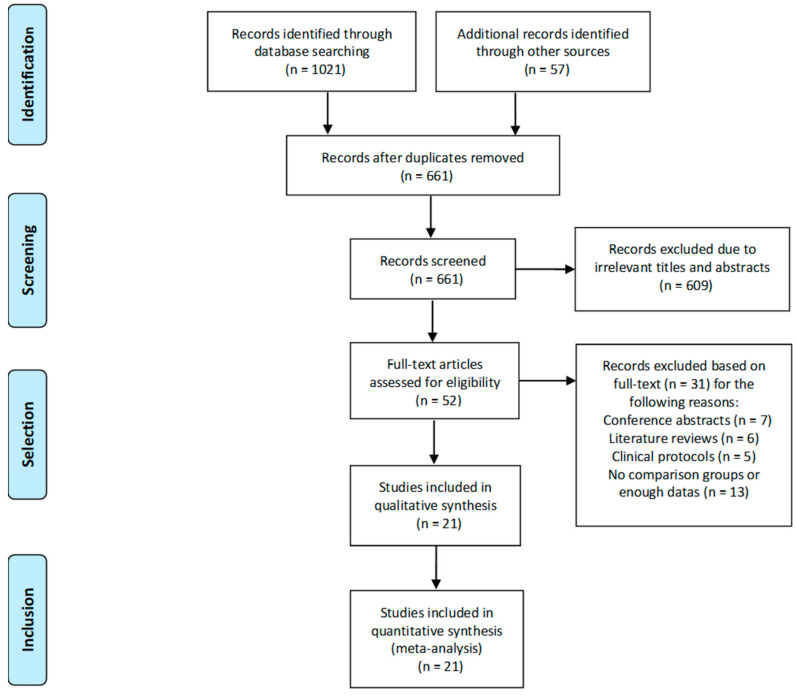
Selection process of the meta-analysis.

A total of 21 articles were included in this meta-analysis. A summary of the specific selection process is presented in Figure 1, according to the recommended PRISMA flow diagram.

A total of 1665 participants were included in the 21 eligible articles of this meta-analysis. Among these included studies, 14 studies focused on healthy older adults and 7 studies focused on older adults with mild cognitive impairment. All participants were older than 50 years old. The mean age of the participants ranged from 67.0 to 77.9, except for one study, which did not give an exact mean age [41]. About 69% of participants were female, excepted for 2 studies, which did not report the gender [41,42]. Sample size ranged from 10 to 261.

Nine studies adopted combined physical and cognitive interventions simultaneously and 12 studies adopted sequential interventions. The simultaneous integrated physical and cognitive interventions consisted of dual-tasks (*n* = 4), exergames (*n* = 2), tai chi (*n* = 2), or virtual reality (*n* = 1). In terms of comparison groups, six studies included four comparison groups, three studies included three comparison groups, and 12 studies included two comparison groups. In the studies that included a comparison to a control group, eight of the studies used an active control group design and the main forms were health education activities and static stretching. Table 1 illustrates the main characteristics of the included articles.

The study quality scores ranged from 7 to 12; 16 studies were supposed to be of high quality, while five studies were considered low quality. The results of Delphi list are presented in Table 1 and Supplementary Materials Table S1

### 3.2. Combined Interventions vs. the Control Group

**Figure 2:**
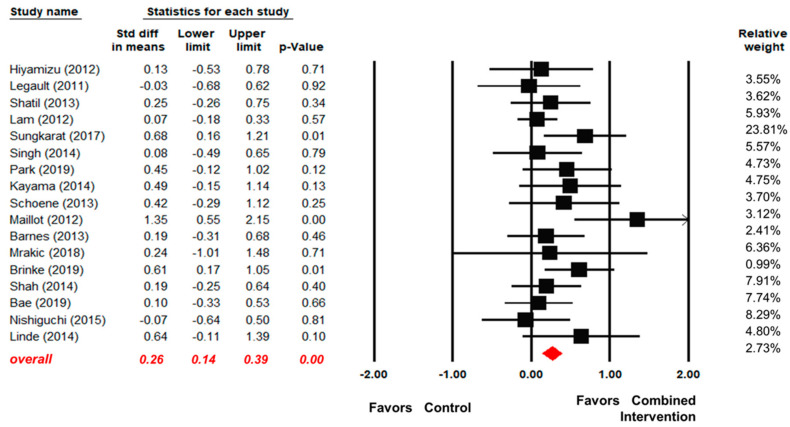
Forest plot for the effect sizes of the combined interventions compared to the control.

A total of 17 articles compared the EFs of older adults between a combined intervention group and a control group [34,41,42,43,44,45,46,47,48,49,50,51,52,53,54,55,56]. As shown in Figure 2, the effect of the combined interventions was significantly better than that of the control group (standardized mean difference (SMD) = 0.26, 95% confidence interval (CI) [0.14, 0.39], *p* < 0.01). The heterogeneity test results revealed no significant heterogeneity between the two groups (Q (16) = 19.61, I^2^ = 18.42, *p* = 0.24).

### 3.3. Combined Interventions vs. Cognitive Intervention Alone

**Figure 3:**
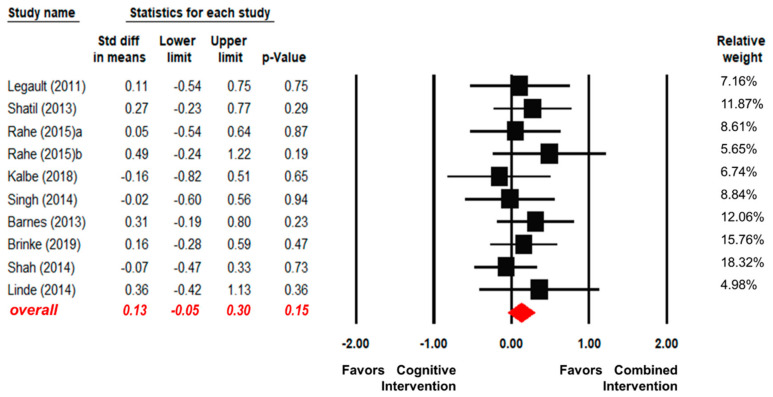
Forest plot for the effect sizes of the combined interventions compared to the cognitive intervention alone.

Ten articles reported the effects of the combined interventions and the cognitive intervention alone [25,34,39,44,47,50,52,53,56,57]. As shown in Figure 3, there were no significant differences between the combined interventions and the cognitive intervention alone in terms of overall EFs (SMD = 0.13, 95% CI [−0.05, 0.30], *p* = 0.15). There was also no significant heterogeneity found in the fixed-effect model across articles (Q (9) = 4.07, I^2^ = 0, *p* = 0.91).

### 3.4. Combined Interventions vs. Physical Exercise Intervention Alone

**Figure 4:**
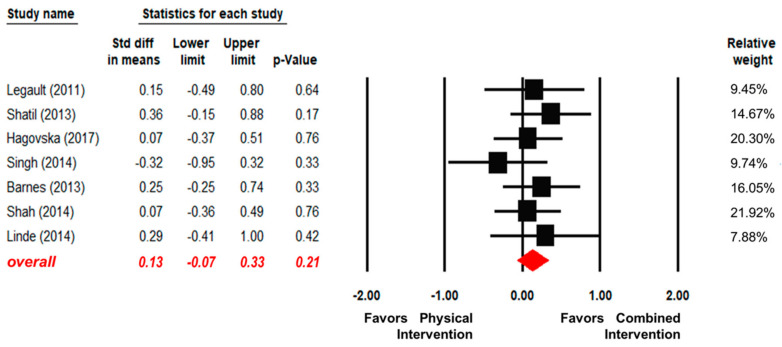
Forest plot for the effect sizes of the combined interventions compared to the physical intervention alone.

Seven articles reported the effects of the combined interventions and the physical intervention alone [34,44,47,50,53,56,58]. As shown in Figure 4, there were no significant differences between the combined intervention groups and the physical exercise intervention alone groups (SMD = 0.13, 95% CI [−0.07, 0.33], *p* = 0.21). In addition, no significant heterogeneity across articles was found (Q (6) = 3.25, I^2^ = 0, *p* = 0.78).

### 3.5. Sensitivity Analyses

**Figure 5:**
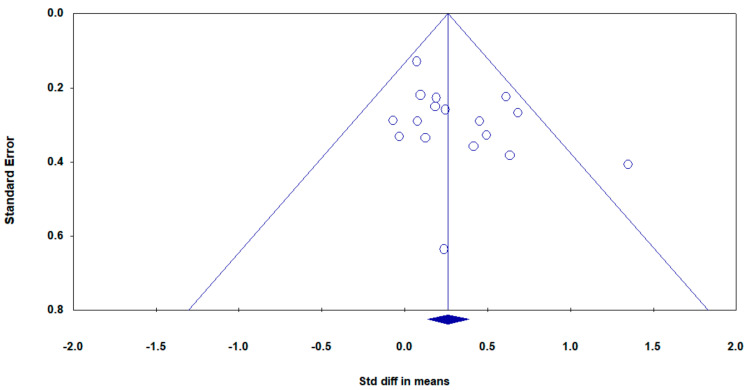
Funnel plot of the combined intervention group vs. the control group.

Funnel plots were used to detect a potential publication bias for the following comparisons: combined interventions vs. control, combined interventions vs. cognitive intervention alone, and combined interventions vs. physical exercise intervention alone. The results revealed that there was a risk of publication bias for the combined interventions vs. control (Figure 5), but not for the other two groups (data not shown). Sensitivity analysis was performed to identify possible outliers for the comparison between the combined interventions and the control group (mean SMD effect size ±3SD). After excluding the one outlier study, there was still a significant difference in the effects between the combined intervention group and the control group (SMD = 0.23, 95% CI [0.11, 0.36], *p* < 0.01).

### 3.6. Effect of Combined Interventions on Sub-Functions of EFs

As shown in Table 2, compared to the control group, the combined interventions generated significant differences in response inhibition (SMD = 0.29, 95% CI [0.10, 0.48], *p* < 0.01), set-shifting (SMD = 0.23, 95% CI [0.10, 0.37], *p* < 0.01), complex EFs (SMD = 0.34, 95% CI [0.13, 0.56], *p* < 0.01), and overall performance (SMD = 0.27, 95% CI [0.17, 0.37], *p* < 0.01). However, the results revealed significant heterogeneity in response inhibition (Q = 20.35, *p* < 0.01, I^2^ = 65.60), complex EFs (Q = 19.27, *p* < 0.01, I^2^ = 63.67), and overall performance (Q = 58.55, *p* < 0.01, I^2^ = 52.18). Compared to the cognitive intervention alone, significant effects of the combined interventions were found for set-shifting (SMD = 0.28, 95% CI [0.04, 0.52], *p* < 0.01) and overall performance (SMD = 0.13, 95% CI [0.01, 0.25], *p* = 0.03), but not for the other two functions. Compared to the physical intervention alone, a significant effect of the combined interventions was only observed for set shifting (SMD = 0.26, 95% CI [0.01, 0.52], *p* = 0.04), but not for the other sub-functions.

### 3.7. Moderator Analyses

To explore the impacts of potential moderator variables on EFs, the following moderator variables were selected: cognitive status of the participants, mode of combination, intervention length, intervention frequency, session length, third component, control group, and methodological quality. Because significant group differences were found only in the comparison of the combined intervention group vs. the control group, analysis of the potential moderator variables was performed only for this comparison.

Table 3 summarizes the results of the potential moderator variable analysis of the combined intervention group compared with the control group. The results showed that the effect of the combined interventions was influenced by the intervention length, which was divided into three subgroups, and the results of the heterogeneity test showed that the combined intervention effects of the three subgroups were marginally different (*p* = 0.09, Q = 4.92). The results showed that the combined interventions had a significant effect on the medium (SMD = 0.37, 95% CI [0.17, 0.57], *p* < 0.01) and short (SMD = 0.44, 95% CI [0.12, 0.75], *p* < 0.01) intervention lengths, but no difference was found for the long intervention lengths. Both the sequential (SMD = 0.27, 95% CI [0.09, 0.44], *p* < 0.01) and simultaneous (SMD = 0.26, 95% CI [0.08, 0.44], *p* < 0.01) combined intervention forms produced significant intervention effects compared with the control group, indicating that different combined intervention strategies may have beneficial effects.

In terms of the cognitive status, the combined interventions had a significant effect on both the healthy (SMD = 0.33, 95% CI [0.16, 0.50], *p* < 0.01) and the mild cognitive impairment (SMD = 0.19, 95% CI [0.01, 0.37], *p* = 0.04) populations, but a larger effect size was generated in the healthy population and the intervention effect was more significant. We also found similar results for the control group. The combined interventions had a positive effect in both the active (SMD = 0.27, 95% CI [0.11, 0.43], *p* < 0.01) and passive (SMD = 0.25, 95% CI [0.05, 0.45], *p* = 0.02) control groups, but the effect was more significant in the active control group. Among the moderator variables, the effect of the combined interventions was significant for only one subgroup. For instance, in terms of the frequency and session duration, the combined interventions had a significant effect on interventions with a low frequency (SMD = 0.28, 95% CI [0.14, 0.41], *p* < 0.01) and a medium duration (SMD = 0.41, 95% CI [0.22, 0.61], *p* < 0.01). In terms of the study quality, there was a significant cognitive gain effect with high-quality studies (SMD = 0.26, 95% CI [0.12, 0.39], *p* < 0.01).

## 4. Discussion

Twenty-one studies were included in this meta-analysis to explore the effect of the combined physical and cognitive interventions on EFs in older adults. The results showed that the combined interventions produced a significantly greater effect compared with the control group in overall EFs. Furthermore, the effects of moderating variables were further explored in this meta-analysis, such as subject characteristics, intervention plan, and study quality.

The present meta-analysis showed that the combined interventions had a significant effect on the overall EFs, compared with that of the control group. While compared with the physical or cognitive intervention alone, no significant differences were found. This result is slightly different from previous meta-analyses that focused on the overall cognitive functions in older adults. They found a significant cognitive advantage of the combined interventions compared with both the control group and the exercise intervention alone group, while no significant difference was found between the combined interventions and cognitive intervention alone [59,60]. The different choice of outcome measures may account for the controversial findings. Our study focused on the effects of combined physical and cognitive interventions on specific EFs in older adults, rather than the overall cognitive functions as in previous studies. Considering the above differences, this study explored the effect of the combined interventions on EFs because different cognitive functions seem to be affected by different types of interventions. This is also one of the important considerations for why the sub-functions of EFs were analyzed in this study.

Although no significant effect was found between the combined and single intervention groups, the effect size reached 0.13 for both the exercise and cognitive intervention alone groups, indicating that the combined interventions still had a weak advantage over the single intervention. Of the studies analyzed, three studies [25,47,53] reported a negative effect for the comparison with the cognitive group and one study [47] reported a negative effect for the comparison with the physical activity group. It is assumed that the duration and intensity of the intervention may account for the negative results. First, the intervention time was too short in these studies. The duration of the intervention moderates the effect. Walsh et al. compared long-term tai chi training with short-term tai chi training and found that short-term tai chi training did not significantly improve cognitive functions in adults [61]. Second, the high-intensity intervention may cause excessive stress and cognitive fatigue in older adults, which may affect the effect size of the intervention [62].

The combined interventions produced a significant effect on all of the sub-functions, including response inhibition, set-shifting, and complex EFs of older adults compared with the control group. The significant effect is additional evidence of lifelong plasticity of the human brain [63]. While compared with physical or cognitive intervention alone, the combined interventions only showed a significant effect on set-shifting of EFs. The ceiling effect helps to explain this result [64]. It is reasonable to assume that the sub-functions of the physical or cognitive intervention group alone probably improved to a ceiling level of the older population. Another possibility is the limitation of approaches aimed to separate cognitive sub-functions in the present meta-analysis. There has been intense theoretical debate in the past two decades about the tasks’ impurity, according to which EFs are entangled with other cognitive processes, which might not be easy to distinguish in the experimental tasks [65]. Also, it has been confirmed that the sub-functions of EFs are not completely independent, however, and do seem to share some underlying commonality [4]. 

The results of moderator analysis showed that the larger benefit of the combined interventions relative to that of the controls depended on the intervention length. The use of a medium (12–23 weeks) or short-term (<12 weeks) intervention produced a significant effect, while a benefit was not found for long-term (≥24 weeks) interventions. It seems that an intervention of less than 23 weeks can effectively improve EFs in older adults. Previous meta-analyses also have revealed that a long intervention length is not necessary for older adults to produce a better cognitive effect in physical intervention studies [66,67]. Of note, only five studies in the present meta-analysis adopted a long-term intervention plan because EFs are sensitive to environmental influences in a relatively short time [68] and long-term intervention is difficult to implement in older adults.

In practice, the determination of which type of combination to choose, simultaneous or sequential, is a controversial issue. A meta-analysis has revealed that the simultaneous combination of physical exercise and cognitive training provides significantly larger gains of cognitive functions in older adults [60]. Exercise can lead to a temporary increase in brain-derived neurotrophic factor, which will return to the baseline level after a period of time. Therefore, the use of simultaneous interventions may integrate the neurotrophic effects of exercise in a timely manner [37]. Another meta-analysis has found that both simultaneous and sequential combination interventions can improve cognitive functions in older adults [59]. Recently, a randomized control trial adopted both the simultaneous and sequential combination of stationary cycling and strategy-based memory training in older adults; the results exhibited that simultaneous training improved composite memory, while sequential training improved EFs [69]. That is to say, different types of combination intervention strategies may affect different aspects of cognition. The present meta-analysis mainly focused on EFs; both the simultaneous and sequential combination interventions can result in increased EFs compared to the controls. Our results also showed that the combined intervention strategy has significant advantages in both the active and passive control groups. In terms of the intervention frequency, a low-frequency (≤3 sessions/week) intervention produced a better intervention effect and in terms of the session duration, a medium duration was the best (>30 min to ≤60 min). The combined interventions had significant effects on the EFs of older adults with or without cognitive impairment. Moreover, a positive correlation between the study quality and the effect of the intervention was also found. The effect of the intervention was more significant for the studies with a higher quality.

To analyze the effect of the combined interventions on EFs in older adults, the present meta-analysis included 21 studies. The combined interventions resulted in a larger benefit in the EFs of older adults than that of the controls. However, there is a lack of a superiority of the combined interventions as compared to the cognitive or physical intervention alone. This is different from previous studies [59,60], but it is quite original and interesting. Besides hypothesizing a role of the different choice of outcome measures mentioned above, it could also be speculated that the combined intervention, which was both mentally and physically challenging, could have resulted in excessive stress, less engagement in home or community-based activities, or other changes that inhibited rather than promoted neural plasticity and cognitive benefits [47]. This speculation may also explain why long intervention duration and high intervention frequency did not produce a significant effect. In this respect, sequential combinations require more time on the intervention and may cause more physical and cognitive load than simultaneous combination.

Although the combined intervention strategy was not found to be superior to physical or cognitive intervention alone in the present meta-analysis, the results confirmed that either type of intervention can affect EFs in older adults. Older adults should keep active, either physically or mentally, to prevent cognitive aging. It should be noted that in most of the literature reports analyzed, the combined intervention group was compared with a control group, while only a few studies compared the differences between the combined interventions and cognitive or physical intervention alone. This limitation may be due to the fact that the latter research design requires a larger number of subjects, which makes it difficult to carry out the research. Furthermore, the sample size was too small (fewer than 20 participants in each group) in some studies, which may cause a low reliability of the results. This is the reason why we preformed the current meta-analysis.

## 5. Conclusions

The combined physical and cognitive interventions effectively alleviated the decline of EFs in older adults. The effect of the combined interventions was affected by the length and frequency of the intervention as well as the quality of the research. However, based on the existing literature data, there is not enough evidence to prove that the combined intervention strategy has more advantages than physical exercise or cognitive intervention alone.

## Figures and Tables

**Table 1:** Main Characteristics of the Included Studies.

	Characteristics				Intervention Methods				EFs Measure Tasks	Control Group Activities	Study Quality
Study	Sample Size	Age (Mean)	Cognitive Status	Comparison	Cognitive Intervention	Physical Intervention	Combination Mode	Intervention Plan			
Hiyamizu (2012)	36	>65 (71.6)	Health	CPI vs. CG	memory, visual search, verbal fluency training	Balance exercise	Simultaneous	60 min/session, 2 sessions/week, 12 weeks	Stroop Task, TMT A, TMT B	NO	8
Legault (2011)	73	70–85 (76.4)	Health	CPI vs. PI vs. CI vs. CG	Memory training	Aerobic and flexibility exercises	Separate	50–150 min/session, 3 sessions/week, 24 weeks	2-Back Test, Flanker Task, Task Switching, TMT A, TMT B, Self-Ordered Pointing Task	NO	9
Maillot (2012)	30	65–78 (73.5)	Health	CPI vs. CG	Nintendo Wii game	Wii Sports, Wii Fit, Mario & Sonic on Olympic Games.	Simultaneous	60 min/session, 2 sessions/week, 12 weeks	TMT A, TMT B; Stroop Test, Letter Sets Test, Matrix Reasoning Test, Digit-Symbol Substitution	NO	9
Barnes (2013)	126	≥65 (73.4)	Health	CPI vs. CI vs. PI vs. CG	Visual and auditory processing speed training	Aerobic exercise	Separate	60 min/session, 6 sessions/week, 12 weeks	TMT B, Erikson Flanker Test	Health video education activities	11
Shatil (2013)	122	65–93 (76.8)	Health	CPI vs. CI vs. PI vs. CG	Cognitive game (CogniFit)	Aerobic, strength, and flexibility exercises	Separate	40–45 min/session, 6 sessions/week, 16 weeks	CogniFit neuropsycholog-ical evaluation	Join a book club	7
Nishiguchi (2015)	48	≥60 (73.3)	Health	CPI vs. CG	verbal fluency, cognitive-motor training	Step exercises, stretching, and strength	Simultaneous	90 min/session, 1 session/week, 12 weeks	TMT A, TMT B	NO	9
Rahe (2015)a	68	50–85 (68.4)	Health	CPI vs. CI	Working memory, fluency, inhibition, planning training	Strength, flexibility, coordination, and endurance exercises	Separate	90 min/session, 2 sessions/week, 7 weeks	Regensburger Wort Flüssigkeits-Test, Stroop Test, WAIS-II (DSB)	N/C	8
Rahe (2015)b	30	50–85 (66.7)	Health	CPI vs. CI	memory, attention, EFs training	Strength, flexibility, and balance exercises	Separate	90 min/session, 2 sessions/week, 7 weeks	Complex Figure Test, TMT A, TMT B	N/C	9
Kalbe (2018)	55	50–85 (68.1)	Health	CPI vs. CI	Memory, attention, EFs training	Strength, flexibility, and balance exercises	Separate	90 min/session, 2 sessions/week, 7 weeks	Stroop Test, WAIS-II (DSB),Regensburger Wort Flüssigkeits-Test	N/C	10
Lam (2012)	261	≥65 (77.8)	MCI	CPI vs. CG	Memory, attention training	Tai Chi	Simultaneous	30 min/session, 3 sessions/week, 12 months	TMT B	Stretching exercise	9
Hagovska (2017)	80	≥65 (67)	MCI	CPI vs. PI	Attention, memory, EFs training	Different forms of walking	Simultaneous	30 min/session, 2 sessions/week, 10 weeks	Stroop Test, TMT A	N/C	10
Sungkarat (2017)	66	≥60 (67.9)	MCI	CPI vs. CG	Memory, attention training	Tai Chi	Simultaneous	50 min/session, 3 sessions/week, 12 weeks	TMT A, TMT B	Education activities	9
Mrakic (2018)	10	≥65 (73.3)	MCI	CPI vs. CG	Memory, visuospatial ability training	Aerobic exercise	Simultaneous	40–45 min/session, 3 sessions/week, 6 weeks	TMT A	NO	8
Singh (2014)	86	55–89 (70.1)	MCI	CPI vs. CI vs. PI vs. CG	Memory, attention, EFs, cognitive processing speed training	Resistance training	Separate	60–100 min/session, 2 sessions/week, 24 weeks	WAIS-III, Controlled Oral Words Association Test	Stretching and education activities	12
Park (2019)	49	>60 (71.6)	MCI	CPI vs. CG	Phrase play, memory play, arithmetic training	Aerobic, balance, stretching	Simultaneous	110 min/session, 2 sessions/week, 24 weeks	Symbol–Digit Substitution Test	NO	10
Kayama (2014)	41	≥65	Health	CPI vs. CG	Placement problem solving (Sudoku)	Tai chi	Simultaneous	75–80 min/session, 1 session/week, 12 weeks	TMT A, TMT B	Strength and balance training	7
Brinke (2020)	124	65–85 (72.4)	Health	CPI vs. CI vs. CG	Cognitive game (Fit Brains)	Brisk walking	Separate	60 min/session, 3 sessions/week, 8 weeks	DCCS, Flanker Test, TMT A, TMT B, Stroop Test	Regular activities	9
Schoene (2013)	32	≥65 (77.9)	Health	CPI vs. CG	Attention training	Step training	Simultaneous	15–20 min/session, 2–3 sessions/week, 8 weeks	TMT A, TMT B	Regular activities	10
Linde (2014)	55	60–75 (67.1)	Health	CPI vs. CI vs. PI vs. CG	Short-term memory, information processing speed, logical reasoning training	Aerobic, endurance, and strength training	Separate	60–90 min/session, 2 sessions/week, 16 weeks	Leistungs-Prüf-System	NO	11
Shah (2014)	172	60–85 (67.4)	Health	CPI vs. CI vs. PI vs. CG	Auditory-based BFP, Visual-based IP	Walking, strength training	Separate	60 min/session, 10 sessions/week, 16 weeks	Groton Maze Learning, Controlled Oral Word Association Test	NO	9
Bae (2019)	83	≥65 (75.9)	MCI	CPI vs. CG	“KENKOJISEICHI” system	“KENKOJISEICHI” system	Separate	90 min/session, 2 sessions/week, 24 weeks	TMT B	NO	9

MCI: mild cognitive impairment. CPI: combined cognitive and physical intervention. PI: physical intervention. CI: cognitive intervention. CG: control group. TMT A: Trail Making Test A. TMT B: Trail Making Test B. WAIS: Wechsler Adult Intelligence Scale, DSB: Subtest Digit Span Backwards. DCCS: Dimensional Change Card Sort Test. BFP: Brain Fitness Program. IP: Insight Program. KENKOJISEICHI: a system which includes 28 physical activities, 29 cognitive activities, and 44 social activities. N/C: No control group.

**Table 2:** Effect Sizes of the Combined Interventions on Response Inhibition, Set-Shifting, and Complex EFs.

Comparison	Outcomes	No. of Studies	SMD	95% Confidence Interval	I^2^ (%)	Homogeneity Test		
						Q	df	*p*
Combined intervention versus Control group	Response inhibition	8	0.29 **	0.10 to 0.48	65.6	20.35	7	0.005
	Set-shifting	13	0.24 **	0.10 to 0.37	34.03	18.19	12	0.11
	Complex EFs	8	0.34 **	0.13 to 0.56	63.67	19.27	7	0.007
	Overall	29	0.27 **	0.17 to 0.37	52.18	58.55	28	0.001
Combined intervention versus Cognitive intervention	Response inhibition	9	0.09	−0.09 to 0.26	0	5.16	8	0.74
	Set-shifting	5	0.04 *	0.04 to 0.50	0	1.15	4	0.87
	Complex EFs	7	0.07	−0.14 to 0.28	0	2.2	6	0.9
	Overall	21	0.13 *	0.01 to 0.25	0	10.6	20	0.96
Combined intervention versus Physical intervention	Response inhibition	6	0.06	−0.15 to 0.26	33.03	7.47	5	0.19
	Set-shifting	4	0.26*	0.01 to 0.52	0	2.04	3	0.56
	Complex EFs	5	0.21	−0.04 to 0.46	0	3.56	4	0.47
	Overall	15	0.16	0.02 to 0.29	5.43	14.8	14	0.39

* *p* < 0.05, ** *p* < 0.05.

**Table 3:** Moderator Analysis for the Combined Intervention Group vs. the Control Group.

Moderator	Level	No. of Studies	SMD	95% CI	I^2^	Homogeneity Test		
						Q	df	*p*
Mode of combination	Sequential	9	0.27 **	0.10 to 0.44	0	0.01	1	0.94
	Simultaneous	8	0.26 **	0.08 to 0.44	49.53			
Cognitive status	Healthy	11	0.33 **	0.16 to 0.50	23.28	1.22	1	0.27
	MCI	6	0.19 *	0.01 to 0.37	6.74			
Control group	Active	8	0.27 **	0.11 to 0.43	14.52	0.03	1	0.86
	Passive	9	0.25 *	0.05 to 0.45	29.78			
Intervention length	Long (≥24 weeks)	5	0.11	−0.07 to 0.29	0	4.92	2	0.09
	Medium (12–23 weeks)	8	0.37 **	0.17 to 0.56	39			
	Short (<12 weeks)	4	0.44 *	0.12 to 0.75	0			
Frequency	High (>3 sessions/week)	3	0.21	−0.07 to 0.48	0	0.2	1	0.66
	Low (≤3 sessions/week)	14	0.28 *	0.14 to 0.41	32.92			
Session duration	Long (>60 min)	7	0.20	−0.02 to 0.41	0	4.13	2	0.13
	Medium (>30 to ≤60 min)	8	0.41 **	0.22 to 0.61	30.47			
	Short (≤30 min)	2	0.11	−0.13 to 0.35	0			
Study quality	High (≥9 scores)	13	0.26 **	0.13 to 0.39	36.65	0.01	1	0.91
	Low (<9 scores)	4	0.28	−0.05 to 0.61	0			

* *p* < 0.05, ** *p* < 0.05.

## Supplementary Materials

The following are available online at https://www.mdpi.com/1660-4601/17/17/6166/s1, Table S1: Delphi list.
